# What do we know about the sleep effects of caffeine used to treat apnoea of prematurity? A systematic review of the literature

**DOI:** 10.1186/s40348-023-00166-2

**Published:** 2023-09-18

**Authors:** Ana Renata Pinto de Toledo, Higor Arruda Caetano, Jovito Adiel Skupien, Carina Rodrigues Boeck, Humberto Fiori, Rosane Souza da Silva

**Affiliations:** 1 Laboratório de Neuroquímica e Psicofarmacologia, Escola de Ciências da Saúde e da Vida, Pontifícia Universidade Católica do Rio Grande do Sul, Porto Alegre, RS Brazil; 2 Programa de Pós-Graduação em Pediatria e Saúde da Criança, Escola de Medicina, Pontifícia Universidade Católica do Rio Grande do Sul, Porto Alegre, RS Brazil; 3 Mestrado em Ciências da Saúde e da Vida, Universidade Franciscana, Santa Maria, RS Brazil; 4 Programa de Pós-Graduação em Nanociências, Universidade Franciscana, Santa Maria, RS Brazil; 5 Programa de Pós-Graduação em Biologia Celular e Molecular, Escola de Ciências da Saúde e da Vida, Pontifícia Universidade Católica do Rio Grande do Sul, Porto Alegre, RS Brazil; 6 Programa de Pós-Graduação em Neurociências, Instituto de Biologia, Universidade Federal Fluminense, Bloco M, Rua Professor Marcos Waldemar de Freitas Reis, S/N, Campus Do Gragoatá, São Domingos, Niterói, RJ CEP: 24210-201 Brazil

**Keywords:** Methylxanthines, Xanthines, Neurodevelopment, Sleep organisation, Sleep quality, Premature

## Abstract

**Objective:**

Scientific scrutiny has proved the safety and benefits of caffeine to treat apnoea of prematurity (AOP). However, there is no consensus on the effects of this treatment on sleep, especially considering the key role of adenosine and early brain development for sleep maturation. We systematically reviewed studies with sleep as a primary and/or secondary outcome or any mention of sleep parameters in the context of caffeine treatment for AOP.

**Methods:**

We performed a systematic search of PubMed, Web of Science and the Virtual Health Library from inception to 7 September 2022 to identify studies investigating the short- and long-term effects of caffeine to treat AOP on sleep parameters. We used the PIC strategy considering preterm infants as the Population, caffeine for apnoea as the Intervention and no or other intervention other than caffeine as the Comparison. We registered the protocol on PROSPERO (CRD42021282536).

**Results:**

Of 4019 studies, we deemed 20, including randomised controlled trials and follow-up and observational studies, to be eligible for our systematic review. The analysed sleep parameters, the evaluation phase and the instruments for sleep assessment varied considerably among the studies. The main findings can be summarised as follows: (i) most of the eligible studies in this systematic review indicate that caffeine used to treat AOP seems to have no effect on key sleep parameters and (ii) the effects on sleep when caffeine is administered earlier, at higher doses or for longer periods than the most common protocol have not been investigated. There is a possible correlation between the caffeine concentration and period of exposure and negative sleep quality, but the sleep assessment protocols used in the included studies did not have high-quality standards and could not provide good evidence.

**Conclusions and implications:**

Sleep quality is an important determinant of health, and better investments in research with adequate sleep assessment tools are necessary to guarantee the ideal management of children who were born preterm.

**Supplementary Information:**

The online version contains supplementary material available at 10.1186/s40348-023-00166-2.

## Introduction

Caffeine has been used to treat and prevent apnoea of prematurity (AOP) for almost five decades [1, 2]. The work of Barbara Schmidt and the Caffeine for Apnea Group, the Canadian Institute of Health Research (CIHR) and the Australian National Health and Medical Research Council (NHMRR) have revealed the benefits of using caffeine to treat children with AOP. These benefits include the following (i) reduction of the duration of exposure to positive pressure and supplemental oxygen; (ii) reduction of the incidence of bronchopulmonary dysplasia, patent ductus arteriosus and severe retinopathy of prematurity; and (iii) increase in survival without neurodevelopmental disabilities (at 18–21 months of age), such as a lower incidence of cerebral palsy and cognitive delay [3]. Additionally, caffeine has proved to be safe, has a long half-life and is cost effective [4].

The caffeine mechanism of action is mediated by blocking adenosine receptors, especially the A_1_ and A_2A_ adenosine receptors, which show high expression in nervous tissue [5]. The mechanism underlying the benefits of caffeine for AOP treatment are not fully understood. Nevertheless, research has shown that caffeine (i) increases minute ventilation, (ii) increases sensitivity to CO_2_, (iii) increases skeletal muscle tone, (iv) decreases diaphragmatic fatigue and (v) increases the metabolic rate with increased oxygen consumption. Adenosine is a potent modulator of cellular homeostasis and immune and neural function; it plays an important role in health and disease, including sleep maturation and performance [6, 7]. Normal development of the central nervous system is obviously required to preserve neural function later in life; hence, surveillance of any disturbance during early brain development is necessary.

There are relatively few side effects attributed to caffeine used to treat AOP; they include irritability, gastrointestinal intolerance and increased sodium and calcium excretion [8]. There is no standardised protocol for the use of caffeine to treat AOP, but there have been numerous studies investigating the best caffeine dose, exposure time and time to start treatment [9, 10]. Some authors claim that there is currently insufficient evidence to support the administration of higher or earlier doses of caffeine to treat AOP [11]. There is evidence that higher doses of caffeine lead to a higher rate of ventilator removal success and serve as an effective treatment for morbidity related to AOP [12]. There is increasing evidence of worse neurological outcomes in infants with a very low birth weight and a high number of apnoeic episodes. Hence, researchers have evaluated protocols that begin caffeine treatment earlier. The results have shown reductions in the duration of mechanical ventilation, the age at the first successful extubation, the need for oxygen supplementation and the incidence of bronchopulmonary dysplasia [13–15]. On the other hand, adjustments to the dose, period and timing of caffeine treatment in neonates with AOP require close observation because haemorrhage and an increased seizure incidence have been correlated with higher caffeine doses and earlier treatment [16, 17].

One of the most studied effects of caffeine is the disruption of sleep and increase in awareness because adenosine is a modulator of sleep [7]. Sleep is an active process that changes continuously throughout life, with the greatest transition after the first 6 months of life [18]. For this reason, evaluation of the sleep effects of caffeine used to treat AOP is necessary. Conversely, normal development of sleep is dependent on preserved respiratory function and normal brain development [18]. In this way, caffeine treatment reduces respiratory problems reducing sleep interferences in infants. Here, we systematically reviewed studies with sleep as a primary and/or secondary outcome or any mention about sleep parameters in the context of caffeine used to treat AOP.

## Material and methods

Before beginning this systematic review, the study protocol was first registered in the International Prospective Register of Systematic Reviews (PROSPERO) under protocol number CRD42021282536. The review was written in accordance with the Preferred Reporting for Systematic Reviews and Meta-Analysis (PRISMA) guidelines.

### Sources of information

Between 30 November 2021 and 1 December 2021, PubMed (https://pubMed.ncbi.nlm.nih.gov), Web of Science (https://www.webofscience.com/wos/woscc/basic-search) and the Virtual Health Library (https://bvsalud.org/) were searched for relevant studies. The DeCS/MeSH terms were CAFFEINE OR XANTHINE OR METHYLXANTHINE AND PRETERM OR NEWBORN OR NEONATAL OR PREMATURE. The order of operation was (CAFFEIN* OR XANTHIN* OR METHYLXANTHIN*) AND (PRETERM* OR NEWBORN* OR NEONAT* OR PREMATUR*). On 7 September 2022, the search was repeated. The details for each search strategy are included in the Supplementary Material.

### Study selection and the PIC strategy

After searching the databases, the studies were selected with the following sequence: (i) exclusion of duplicates, (ii) title analysis, (iii) analysis of abstracts, (iv) analysis of the complete manuscript and (v) analysis of the cited references. These steps were performed by two independent reviewers (A. R. P. T. and H. A. R.), followed by a final review by the senior researcher (R. S. S.). Only original studies were considered.

The PIC strategy was Population, preterm newborns < 37 weeks; Intervention, caffeine citrate therapy; and Comparison, preterm infants < 37 weeks old who had received no intervention or any intervention other than caffeine. Only studies written in English, Portuguese or Spanish were considered.

### Eligibility criteria

Considering the search strategy, the eligible articles were studies in human preterm newborns at < 37 gestational weeks, exposed to caffeine and with any parameter able to assess sleep. Original studies, including experimental studies, epidemiological studies, cohort/longitudinal studies, cross-sectional studies, randomised controlled trials (RCTs), case–control study and case studies, were eligible. Animal studies, in vitro studies, studies that did not measure the outcomes of this systematic review, data from individuals with neurological sequelae and non-original studies (reviews, abstracts and annals of scientific meetings) were excluded.

### Data extraction

Two independent reviewers (A. R. P. T. and R. S. S.) extracted data from the eligible studies. The extracted data included the type of study, sample comparison, the gestational age at birth, the gestational age at which the newborn was exposed to caffeine, the number of infants submitted to the intervention, sex, the caffeine dose applied, the time of exposure to caffeine and the age at which the sleep outcome was collected. In addition, the types of sleep monitoring, the main outcomes, how the data were collected, the country of origin and the year of publication were recorded.

### Data synthesis and risk-of-bias evaluation

The collected outcomes were grouped as best as possible according to the design of each study, namely observational and interventional (RCTs and follow-ups). The following data were synthesised: bibliographic information, the study design, the main objective, the type of comparison, the population characteristics, the intervention characteristics (gestational age of exposure, caffeine exposure regimen and total time of exposure) and outcomes related to sleep and caffeine exposure (the period of sleep evaluation, the type of sleep evaluation and the main results).

The risk of bias of each study was assessed with the Cochrane RoB 2 Checklist (2021 version) [19] or the Newcastle–Ottawa scale [20].

## Results

**Fig. 1:**
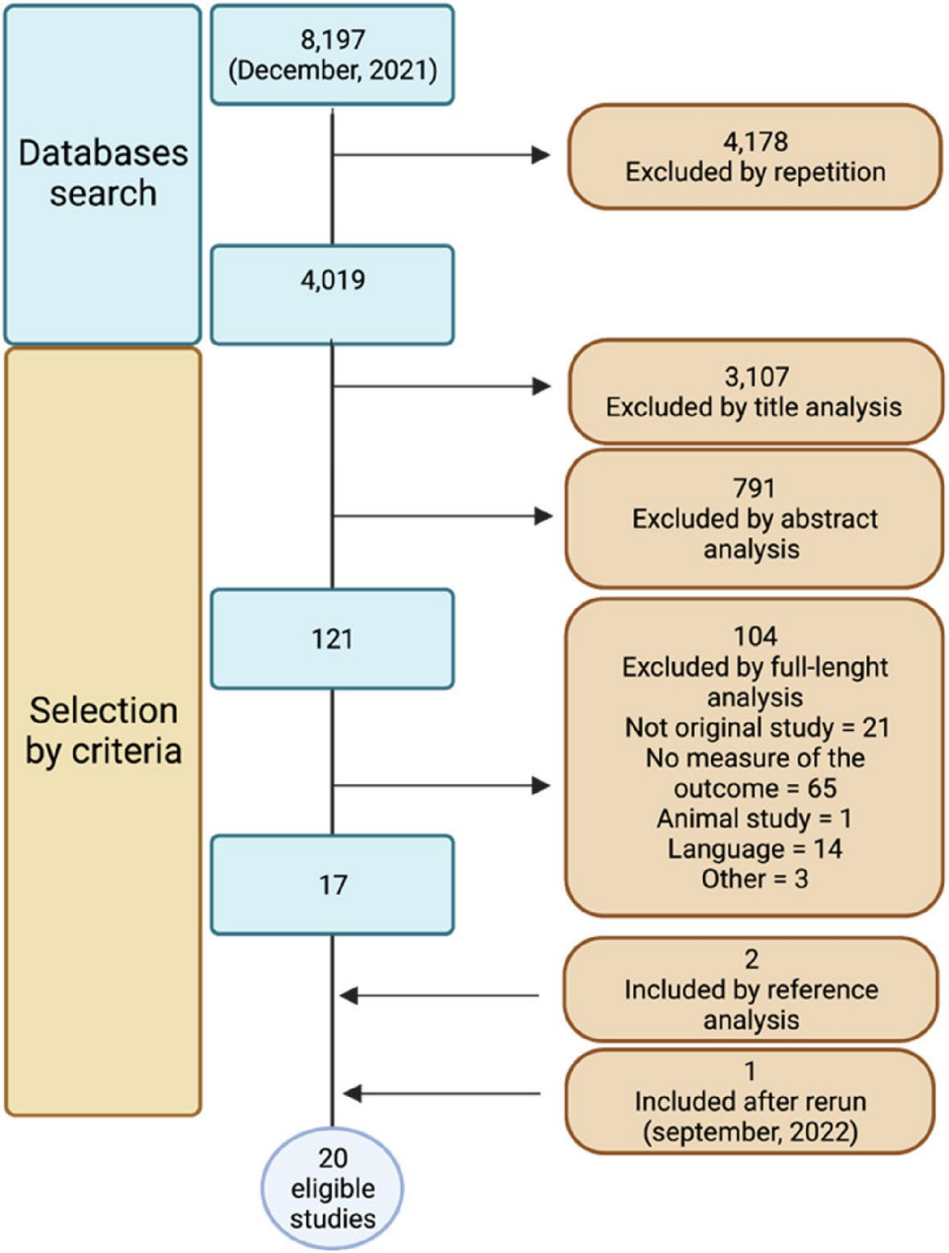
Flow chart of the study selection. Only studies written in English, Portuguese and Spanish were considered

After applying the inclusion criteria, 20 studies were eligible (Fig. 1). We could not perform a meta-analysis of the selected studies because the authors had used a wide array of strategies to assess sleep parameters. In addition, we identified uncertainties regarding the acquisition of sleep parameters. However, we found interesting data from RCTs, follow-ups of RCTs and observational studies, which we describe narratively. Table 1 describes main features of eligible studies.

**Table 1:** Main characteristics of the eligible studies

Ref	Study design and main aim	Comparison	Population	Caffeine exposure regimen	Sleep results related to caffeine exposure
[21]	This randomised clinical trial evaluated whether neonatal caffeine use resulted in long-term abnormalities in sleep architecture and breathing during sleep	Caffeine *versus* placebo	Children aged 5–12 years	20 mg kg^−1^ loading dose of caffeine citrate followed by a daily maintenance dose of 5 mg kg^−1^ during the first 10 days of life	**Actigraphy**: no difference between groups **Polysomnography**: total sleep time was longer in the caffeine group compared with the placebo group, but there was no difference in sleep efficiency or sleep architecture between the groups **Questionaries**: no significant difference between the groups, but caregivers in the caffeine group thought that their child needed more sleep than control caregivers (*p* = 0.007) No long-term effects of neonatal caffeine therapy on objective and subjective measures of sleep at school age
[22]	This study evaluated data from a large randomised clinical trial with preterm infants to evaluate the validity of commonly used actigraphy compared with polysomnography	Caffeine and placebo groups pooled	Children aged 5–12 years	20 mg kg^−1^ loading dose of caffeine citrate followed by a daily maintenance dose of 5 mg kg^−1^ during the first 10 days of life	**Actigraphy**: no indication of a caffeine effect **Polysomnography**: no indication of a caffeine effect
[23]	This study examined data from a randomised clinical trial to compare sleep–wake patterns in children who were born preterm in Australia and Canada and determined cultural differences in the relationship between parental perception of sleep and actual sleep behaviours	Caffeine and placebo groups pooled	Children aged 5–12 years	20 mg kg^−1^ loading dose of caffeine citrate followed by a daily maintenance dose of 5 mg kg^−1^ during the first 10 days of life	**Actigraphy**: no indication of a caffeine effect **Sleep diaries**: no indication of a caffeine effect
[24]	This study examined data from a randomised clinical trial to determine whether children who were born preterm would have a high prevalence of restless legs syndrome and periodic limb movement disorder	Caffeine and placebo groups pooled	Children aged 5–12 years	20 mg kg^−1^ loading dose of caffeine citrate followed by a daily maintenance dose of 5 mg kg^−1^ during the first 10 days of life	**Polysomnography**: preterm births had a high prevalence of restless legs syndrome between the ages of 5 and 12 years Caffeine use does not appear to contribute to this disorder
[25]	This study examined data from a randomised clinical trial to determine perinatal factors associated with obstructive sleep apnoea syndrome at school age	Caffeine and placebo groups pooled	Children aged 5–12 years	20 mg kg^−1^ loading dose of caffeine citrate followed by a daily maintenance dose of 5 mg kg^−1^ during the first 10 days of life	**Polysomnography**: no indication of a caffeine effect
[26]	This study examined data from a longitudinal randomised study to determine risk factors with the potential to affect short-term neurobehavioural and sleep outcomes in preterm infants born	Preterm infants accompanied during caffeine exposure	Infants aged 32–36 weeks of gestational age	No specific data available about the caffeine regimen	**Videotape recordings**: caffeine was significantly related to less quiet sleep Caffeine use was associated with lower scores for alertness and orientation, motor, irritability, cry quality, popliteal angle and scarf sign and higher per cent time asleep
[10]	This randomised clinical trial aimed to establish the most effective and best tolerated dose of caffeine citrate for the prevention of intermittent hypoxaemia in late preterm infants	Caffeine *versus* placebo	Infants aged 38–40 weeks of gestational age	Loading dose (10, 20, 30, or 40 mg kg^−1^) followed by 5, 10, 15, or 20 mg kg^−1^/day^−1^ caffeine citrate	**Oximetry and questionnaires**: no indication of a caffeine effect
[27]	This observational study aimed to evaluate sleep organisation in neonates hospitalised in a neonatal intensive care unit	Caffeine *versus* no caffeine	Infants aged 33.9 ± 0.1^a^ weeks of postmenstrual age	20 mg kg^−1^ loading dose of caffeine citrate followed by a daily maintenance dose of 5 mg kg^−1^	**Polysomnography**: sleep variables were similar before and after the caffeine dose (or the time of dose in controls)
[28]	This observational study investigated the characteristics and effects of sleep stage, supplemental oxygen and caffeine on periodic breathing and apnoea of prematurity in preterm infants	Baseline *versus* before caffeine	Infants aged 35.7^b^ weeks of gestational age	20 mg kg^−1^ loading dose of caffeine citrate followed by a daily maintenance dose of 5 mg kg^−1^	**Polysomnography**: caffeine reduced the median sleep time on periodic breathing by 91% (*p* < 0.001) The average number of desaturations per hour with caffeine decreased from 38 to 24
[29]	This observational study investigated short-term effects of caffeine on sleep in late preterm infants using polysomnography	Baseline *versus* before caffeine	Infants aged 35.7^b^ weeks of gestational age	20 mg kg^−1^ loading dose of caffeine citrate followed by a daily maintenance dose of 5 mg kg^−1^	**Polysomnography**: caffeine reduced the number of apnoeic events (*p* < 0.0001) A high caffeine loading dose of 20 mg kg^−1^ did not affect sleep stage distribution, sleep efficiency, frequency of sleep stage transitions, rapid eye movement sleep, or the number of spontaneous awakenings
[30]	This observational study determined whether apnoeic preterm infant currently treated with methylxanthines develops evidence of sleep deprivation from cumulative arousal and motor activational effects	Caffeine *versus* no caffeine	Infants aged 33.2 ± 1.9^a^ weeks of postmenstrual age	Not clear — standard regimens of caffeine based on a blood range of 13.1–29.2 µg ml^−1^	**Video recordings and actigraphy**: caffeine groups exhibited less wakefulness than untreated infants according to per cent wakefulness, the number of brief awakenings, sustained awakenings and lower scores on the composite arousal index Time of exposure to methylxanthine was associated with a linear increase in wakefulness and motor parameters, especially caffeine, promoting sleep fragmentation The results suggest that chronic treatment with methylxanthine appears to produce sleep deprivation secondary to the stimulating action of methylxanthine on the arousal system
[31]	This observational study aimed to evaluate sleep problems in preterm infants (at 6 months of age) and compare the sleep of preterm infants with that of full-term infants	Mature infants *versus* preterm infants treated with caffeine	Infants aged 6 months	20 mg kg^−1^ loading dose of caffeine citrate followed by a daily maintenance dose of 5 mg kg^−1^	**Brief Infant Sleep Questionnaire, actigraphy and polysomnography**: no indication of a caffeine effect
[32]	This observational study aimed to identify the components of the neonatal medical history associated with childhood sleep-disordered breathing in children who were born preterm	Children with or without sleep-disordered breathing treated with caffeine	Children aged 8–11 years	No specific data available about the caffeine regimen	**Data collection from chart review of data from hospital and cardiorespiratory recording**: xanthine was associated with a more than twofold increase in the chance of sleep-disordered breathing, which was slightly attenuated after adjusting for race Xanthine exposure was associated with childhood sleep-disordered breathing in unadjusted analyses, although it is not proof of a causal association between neonatal xanthine exposure and childhood sleep-disordered breathing
[33]	This observational study investigated how caffeine treatment affects sleep–wake behaviour in preterm neonates	Caffeine *versus* no caffeine	Infants aged 30.6^b^ weeks of gestational age	Not clear — caffeine concentration simulated by mathematical model	**Videographic recordings**: wakefulness increased, and active sleep decreased as the caffeine concentration increased, while quiet sleep remained unchanged
[34]	This observational study examined patterns of behavioural states of preterm newborns before and after nursing interventions	Caffeine *versus* no caffeine	Infants aged 32.7 ± 1.3^a^ weeks of gestational age	No specific data available about the caffeine regimen	**Videographic recordings**: significantly more waking hours before the intervention for the newborns in the control group compared with the newborns receiving xanthine (*p* = 0.046) The frequency of wake bouts for the newborns receiving xanthine increased slightly after nursing interventions The change in wake bouts was significant in the control group, while the before–after difference in wake bouts for the newborns receiving xanthine was not significant (*p* = 0.076)
[35]	This observational study assessed the activity of the peripheral chemoreceptors in relation to sleep stages in preterm neonates treated or not treated with caffeine	Caffeine *versus* no caffeine	Infants aged 36.1 ± 0.8^a^ weeks of post-conceptional age	4.0 ± 0.5 mg kg^−1^ day^−1^ of caffeine	**Electroencephalograms, eye movement (transducer), actigraphy, visual observations and hyperoxia test**: regarding sleep parameters, there was no effect of caffeine either on total sleep time or on the durations of sleep stages expressed as a percentage of total sleep time
[36]	This observational study examined the development of respiration during the preterm and early post-term periods and the effects of biological variables of sleep	Preterm infants followed for 1–3 months	Infants up to 43 weeks of post-conceptional age	No specific data available about the caffeine regimen	**Sleep visual observation**: there was an interaction between postconceptional age and methylxanthine (theophylline or caffeine) treatment for the variability of respiration rate in active sleep
[37]	This observational study examined the development of sleeping and waking during the preterm and early post-term periods and the effects of infant health and environmental characteristics	Preterm infants followed until hospital discharge or 44-week post-conceptional age	Infants up to 44 weeks of post-conceptional age	No specific data available about the caffeine regimen	**Sleep visual observation, electroencephalograms and respiration record**: covariates had minor effects on sleep–wake parameters Greater respiration regularity in active sleep occurred during treatment with methylxanthines
[38]	This observational study investigated the effects of caffeine on respiratory functions and cerebral activity and the long-term effects on the respiratory system and encephalographic maturation of preterm infants	Caffeine *versus* no caffeine	Infants aged 36 weeks of postmenstrual age	Loading dose of 20 mg kg^−1^ of caffeine	**Electroencephalograms and sleep state stages**: caffeine increased the cerebral cortical activity of preterm infants during infusion (amplitude-integrated electroencephalography continuity [*p* = 0.002] and arousability after 30 min [*p* = 0.000]) and results in cerebral cortical maturation at 36 weeks
[39]	This observational study examined possible effects of incubator covers on sleep patterns in stable preterm infants	Preterm infants using covers *versus* no covers	Infants aged 32–34 weeks of post-conceptional age	No specific data available about caffeine regimen	**Electroencephalograms**: there were no effects on quiet sleep (duration, intervals, or per cent) for infants treated with theophylline/caffeine

^a^Mean ± standard deviation

^b^Median

### RCTs and follow-ups of RCTs

Of the 20 included studies, there was one RCT [10] and seven follow-ups [21–26] that evaluated sleep parameters in interventional studies considering caffeine exposure in children who were born premature.

#### Long-term assessments

Marcus et al. [21] found no long-term effects of caffeine exposure during the first 10 days of life on the primary outcome of total sleep time detected with actigraphic analysis of 5–12-year-old children. The authors also evaluated bedtime, sleep onset latency and wakefulness after sleep onset as secondary outcomes; they were also unaffected. On the other hand, their polysomnographic assessment showed greater total sleep time when comparing infants treated with caffeine with infants treated with placebo, while there was no difference in sleep efficiency and architecture. Although the periodic limb movements during sleep were higher in the caffeine group compared with the placebo group, this difference vanished when considering covariates [21].

Meltzer et al. [22] carried out a study to validate actigraphy compared with polysomnography for sleep assessment in a pooled sample of 5–12-year-old children from the caffeine and placebo groups reported by Marcus et al. [21]. While the objective of this study was to evaluate technologies and their suitability for sleep assessment, it is worth mentioning that the authors did not observe an effect in the same sample from the previous study by Marcus et al. [21] — and thus from the CAP study [4]. In other words, Meltzer et al. [22] no longer detected the effects on total sleep time reported by Marcus et al. [21].

Biggs et al. [23], Cielo et al. [24] and Tapia et al. [25] published follow-ups of the CAP study. They considered only infants exposed to caffeine, or they pooled children exposed to placebo and caffeine into a single group. Based on actigraphy and sleep diaries, Biggs et al. [23] suggested that irregular sleep schedules and reduced sleep duration relative to the recommendations are common in 5–12-year-old children who were born preterm in Australia and Canada. They highlighted the lack of a control group as an important limitation of their study. Cielo et al. [24] studied a pooled sample of children exposed to caffeine and placebo from the CAP study considering the presence of periodic limb movements and restless legs syndrome in these 5–12-year-old children who were born preterm. These children had a high prevalence of restless legs syndrome (8.4%) and periodic limb movements (7.8%), detected by means of home polysomnographic assessment [24]. The authors assumed that the lack of a difference in the number of patients with periodic limbic movements between the groups implies that neonatal caffeine administration is not a risk factor for increased limb movement during sleep [24]. Likewise, Tapia et al. [25] collected data from pooled groups exposed to caffeine and placebo in the CAP study and analysed risk factors for obstructive sleep apnoea (OSA) through polysomnographic evaluation. They concluded that caffeine did not increase the incidence of OSA.

#### Assessments while receiving caffeine treatment

Brandon et al. [26] performed a follow-up of the former study from the same group [40] to evaluate the risk factors for short-term neurobehavioural and sleep outcomes in preterm infants through video recording to evaluate cluster scores related to sleep quality. They indicated that the use of caffeine during the assessment was associated with lower scores for alertness and higher scores for time asleep, while the amount of quiet sleep was reduced. More recently, Oliphant et al. [10] performed a short-term analyses of caffeine effects, evaluating intermittent hypoxaemia as a primary outcome and sleep parameters as secondary outcomes. There were no effects on sleep based on questionnaires answered by parents of premature newborns who received a loading dose of 10, 20, 30 or 40 mg kg^−1^ followed by 5, 10, 15 or 20 mg kg^−1^ day^−1^ equivolume enteral caffeine citrate as a maintenance dose [10].

### Observational studies

We identified 13 observational studies regarding the effects of caffeine used to treat AOP on sleep parameters [27–39]. The authors of these studies applied a wide array of methods, including polysomnography, actigraphy, visual observations and electroencephalography.

#### Long-term assessments

There were two studies that reported long-term assessment regarding the effects of caffeine used to treat AOP in children aged 6 months [31] and 8–11 years [32] Huang et al. [31] demonstrated that at 6 months of age, preterm infants exposed to caffeine had more sleep problems than full-term infants who did not receive caffeine. They noted significant correlations between actigraphic and polysomnographic evaluations and the Chinese version of the Brief Infant Sleep Questionnaire (BISQ) and sleep diaries [31]. The sleep problems that correlated with actigraphic and polysomnographic evaluation were the nocturnal sleep duration, the number of night awakenings, the daytime sleep duration, the duration of time with mouth breathing and the duration of time with noisy breathing [31]. Hibbs et al. [32] reviewed birth records of preterm neonates and analysed data from cardiorespiratory evaluation and sleep diaries collected at around 10 years of age. They associated the use of xanthines, including caffeine, with a more than twofold increase in the chance of sleep-disordered breathing.

#### Assessments while receiving caffeine treatment and immediately after caffeine discontinuation

We identified observational studies focused on assessments between the first days to up to 12 weeks of life, included analyses during caffeine treatment or close to discontinuation of treatment [27–30, 33–39].

Curzi-Dascalova et al. [27] suggested no differences in sleep organisation between control and caffeine-treated premature infants evaluated with polysomnography during the maintenance phase of the treatment. In a different approach, but also using polysomnography, Seppä-Moilanen et al. [28] compared sleep stage, caffeine and supplemental oxygen effects over periodic breathing, considering baseline and after acute caffeine exposure in a group of premature infants. Caffeine and supplemental oxygen reduced the extent of periodic breathing and the number of periodic breathing related to apnoea, reducing the impact on sleep quality. In a study of the short-term effects of caffeine on sleep in late preterm infant, the same group showed that all the main sleep quality attributes, such as total sleep time and sleep stage transition, remained similar in both study phases after acute caffeine exposure in late preterm infants [29].

Hayes et al. [30] demonstrated by actigraphic and videographic assessments that the cumulative exposure to caffeine in premature infants increases maximum movement bout duration, decreases sleep-related spontaneous movements and diminishes active sleep as the duration of exposure increases when compared with nonexposed infants.

Chardon et al. [35] did not find an effect of caffeine exposure during the first 3 weeks of life on the total sleep time or the sleep stage durations expressed as a percentage of total sleep time using sleep stage scores based on electroencephalograms, eye movement using transducers, body movements by actigraphy, visual observations and the hyperoxia test. Hassanein et al. [38] demonstrated a significant increase in awakening 30 min after caffeine exposure in a prospective observational study.

Symanski et al. [34] reported a significant interaction between xanthine status and measurements before and after awakenings based on video recordings. Their findings indicated that neonates receiving xanthine therapy, including caffeine, responded differently from control neonates regarding wakefulness behaviour during an examination of sleep behaviour states before and after nursing interventions. They also reported that the frequency of awakenings for the newborns who received xanthine increased slightly after the nursing interventions. Koch et al. [33] used video recordings and found that neonates with gestational age ≥ 28 weeks receiving caffeine treatment had an increased fraction of wakefulness, alertness and likely also arousability at the cost of active but not quiet sleep during first 5 days of life. The sleep effects were reinforced as the caffeine concentration increased.

Holditch-Davis et al. [36] found that methylxanthines (theophylline or caffeine), which they considered as a covariate, affected most of the evaluated sleep parameters. Specifically, during theophylline or caffeine treatment or shortly after its discontinuation, infants showed less variability in active sleep, shorter breathing pauses, fewer breathing pauses per hour of active and quiet sleep, less chance of periodic breathing in both sleep states and greater breathing during active sleep under. Using video recordings and electroencephalography, the same group observed sleep–wake states in infants until hospital discharge or 44 weeks post-conceptional age and at one follow-up between 1 and 3 months after term [37]. While receiving treatment, the number of days during which theophylline or caffeine was used contributed to differences between infants from different hospitals and greater regularity in the respiration pattern [37]. Hellström-Westas et al. considered methylxanthine use as a covariate that had no electroencephalographic effects on the duration, interval or percentage of quiet sleep in 9- and 48-day-old infants receiving treatment [39].

### Quality of the included studies

Supplementary Figs. 1 and 2 show a summary of the evaluation of risk of bias. Marcus et al. [21] were the only eligible experimental study rated as low risk of bias because it was the only one to compare placebo versus caffeine-treated infants and that considered sleep parameters as the primary outcome. The lack of separation between caffeine and a nonexposed caffeine group, inadequate methods to measure sleep parameters and uncertainties regarding blinding of investigators and analysis of data related to sleep were the main issues in the other RCTs and follow-ups [10, 22–26].

We rated each most of the included observational studies as low risk of bias based on the Newcastle–Ottawa scale [27–39]. However, three observational studies [32, 36, 39] lacked a nonexposed group, a factor that contributed to high risk of bias in the selection bias parameter of the Newcastle–Ottawa scale. Finally, four studies [28, 29, 35, 38] had uncertainties regarding assessment of the results in the specific parameter ‘assessment of outcome’ of the Newcastle–Ottawa scale.

## Discussion

Of the 20 studies we included, five were follow-up assessments of the long-term effects of children who were born preterm participating in the CAP study [21–25]. The studies that used less accurate tools, such as actigraphy and answers/annotations to questionnaires/sleep diaries from parents, did not find any effects on sleep-related parameters [21, 23]. When a more appropriate tool (polysomnography) was used to assess these long-term effects, there was an increase in total sleep time in caffeine-treated children compared with children treated with placebo, although the authors attributed this result to chance [21]. Additionally, Marcus et al. [21] reported that no other sleep parameter was affected based on polysomnographic evaluation. Other studies using the same group of infants, but pooled into a single group, did not find that caffeine altered sleep-related parameters [22, 24, 25]. These long-term studies used the same source of data, so it is worth mentioning that these results are related to the same caffeine exposure protocol: 20 mg kg^−1^ as a loading dose followed by a daily maintenance dose of 5 mg kg^−1^ beginning during the first 10 days of life.

Two observational studies included long-term assessments of sleep at 6 months [31] and 8–11 years [32] of age. Huang et al. [31] reported an important correlation between data from questionaries, actigraphy and polysomnography, but did not find effects of caffeine on sleep parameters. However, several limitations must be carefully considered — for example they compared preterm infants treated with caffeine with full-term infants and did not consider the impacts of prematurity per se on sleep parameters [31]. On the other hand, Hibbs et al. [32] found an association between xanthine exposure and sleep-disordered breathing, while the diagnosis of AOP was not associated with this disorder. However, this study did not differentiate caffeine data from theophylline data, and there were no adjustments to the data. In the end, the authors called for attention to the potential long-term contribution of xanthine exposure to sleep-disordered breathing in school-age children [32].

Thirteen studies reported the sleep impacts of ongoing caffeine treatment or shortly after discontinuation of caffeine; these studies used different approaches, making it difficult to compare the results [10, 26–30, 33–39] Brandon et al. [26] showed sleep fragmentation and decreased quiet sleep in preterm infants during caffeine exposure in a follow-up of an RCT. These results deserve attention because quiet sleep is the most important sleep stage for brain development. Holditch-Davis et al. [36] reported that infants receiving caffeine treatment had fewer respiratory pauses per hour during quiet sleep. The authors attributed this effect to the direct pharmacological action of methylxanthines during the early post-term period. Meanwhile, observational studies also performed during caffeine exposure did not find an effect on quiet sleep [33, 39] or sleep stage distribution [29, 35].

Caffeine treatment was associated with decreased active sleep [30, 33], with a positive correlation with increasing caffeine concentration. There were also correlations between increasing caffeine concentration and exposure time with wakefulness and motor parameters, promoting sleep fragmentation [33, 34]. On the other hand, Oliphant et al. [10] reported no effect on sleep in a recent RCT when using nonconventional loading and maintenance doses of caffeine to treat AOP. Unfortunately, none of these studies used the gold standard tool for sleep assessment: polysomnography [41]. Nevertheless, when Seppa-Moilanen et al. [28, 29] used polysomnography for observational assessments of a small group of infants being treated with caffeine, they did not find effects on sleep stages, but caffeine-treated infants showed a reduction in the extent of periodic breathing and the number of periodic breathing-related apnoeic episodes. Curzi-Dascalova et al. [27] also used polysomnography and found similar results for sleep variables before and after caffeine application. The relationship between sleep quality and apnoeic event frequency and duration is a direct effect of the ability of caffeine to improve the respiratory response and appears to improve sleep quality [29].

There are several limitations of the included studies that need to be addressed. The most important limitations are the use of different coding for sleep stages, the use of different parameters to evaluate sleep and differences in the types and adequacy of tools to assess sleep. These differences are probably because (i) most studies did not address sleep directly as a primary outcome and (ii) polysomnography is complex and not available for all studies. These factors emphasise the need for more studies in this area. Finally, only one study [10] investigated sleep parameters using a non-standard caffeine protocol to treat AOP. The increasing use of non-standard caffeine protocols to treat very young premature infants requires greater attention from the scientific community.

Considering the importance of sleep for quality of life, the finding that the most common protocol for caffeine to treat AOP did not have important long-term effects on sleep parameters in RCTs and follow-ups using polysomnography is quite encouraging. Although a higher caffeine dose and earlier treatment start have shown benefits, especially in very young preterm infants, several indicators of quality of life still require investigation [11]. Among the benefits of beginning caffeine treatment earlier are a decrease in the incidence of bronchopulmonary dysplasia, death and bronchopulmonary dysplasia, the need to treat patent ductus arteriosus, the incidence of intraventricular haemorrhage, the need for therapy to address retinopathy of prematurity and the use of postnatal steroids [42, 43]. A mild increase in the maintenance caffeine dose has been linked to reduction in the incidence of apnoea, extubation failure and the duration of mechanical ventilation and possible improvement in early neurodevelopmental outcomes [43–45]. Concerns regarding an increased incidence of seizures, tachycardia and cerebellar haemorrhage have been suggested as impacts of higher doses of caffeine administered earlier than 24 h after birth [16, 17, 43, 46]. However, the lack of good-quality evidence for all outcomes of higher caffeine doses and earlier treatment start [9, 43] calls for further investigation, including long-term neurodevelopmental outcomes and the effects on sleep quality.

## Conclusion

Most of the eligible studies in this systematic review of the literature indicate that when AOP is treated with caffeine administered using a near-standard protocol (20 mg kg^−1^ as a loading dose and 5–10 mg kg^−1^ as a maintenance dose), there are not large effects on key sleep parameters. However, some studies using a standard caffeine protocol to treat AOP or adjustments in onset period and caffeine dose have shown impacts on sleep parameters, but the sleep assessment protocols were not of sufficient quality to produce good evidence. It is also worth mentioning that there has been insufficient research regarding the impacts of earlier and higher doses of caffeine to treat very young preterm infants, especially when evaluating sleep maturation. Sleep quality is a source of health, and better investments in research with adequate tools for sleep assessment are essential to guarantee the ideal management of survivors of prematurity.

### Supplementary Information

**Additional file 1:** Supplementary figures: Summary of risk of bias evaluation. **Supplementary Fig. 1.** Summary of Cochrane Risk of Bias analysis following Sterne and colleagues [19]. **Supplementary Fig. 2.** Summary of Newcastle-Ottawa of Bias analysis. A* and B* = low risk of bias and C and D= high risk of bias following Wells and colleagues [20].

## Data Availability

The datasets used and/or analysed during the current study are available from the corresponding author upon reasonable request.
