# Supplementary material for: What do we know about the sleep effects of caffeine used to treat apnoea of prematurity? A systematic review of the literature
Source: Mol Cell Pediatr. 2023 Sep 18;10:13. doi: 10.1186/s40348-023-00166-2 (PMC10505599; doi:10.1186/s40348-023-00166-2)
Supplement: Supplementary file 1 — Additional file 1: Supplementary figures: Summary of risk of bias evaluation. Supplementary Fig. 1. Summary of Cochrane Risk of Bias analysis following Sterne and colleagues [19]. Supplementary Fig. 2. Summary of Newcastle-Ottawa of Bias analysis. A* and B* = low risk of bias and C and D= high risk of bias following Wells and colleagues [20]. [file 40348_2023_166_MOESM1_ESM.docx]

**Supplementary material**

**Title:** What do we know about the sleep effects of caffeine given to treat apnea of ​​prematurity? A Systematic Review of the Literature

**Authors:** Ana Renata Pinto de Toledo^1;2^; Higor Arruda Caetano^1^; Jovito Adien Skupiel^4^; Carina Rodrigues Boeck^4;5^; Humberto Fiori^2^; Rosane Souza da Silva^1;2;3*^

***Contact details of the corresponding author**

**Present Address of corresponding author:**

Universidade Federal Fluminense  -

Instituto de Biologia - Bloco M - Campus do Gragoatá Depto. de Neurobiologia

Rua Professor Marcos Waldemar de Freitas Reis, s/n,

Campus do Gragoatá – São Domingos

Niterói – RJ   CEP: 24210-201

E-mail: [rosanesouzadasilva@gmail.com](mailto:rosanesouzadasilva@gmail.com)

Search Strategies:

PUBMED:

https://pubmed.ncbi.nlm.nih.gov/?term=(caffein*%20OR%20Xanthin*%20OR%20Methylxanthine)%20AND%20(preterm*%20OR%20newborn*%20OR%20neonat*%20OR%20prematur*)

Web Of Science:

https://www.webofscience.com/wos/woscc/summary/ec0896ad-10c7-4a0e-80a9-925f087afb0d-167a94ac/relevance/1

BVS/MEDLINE:

https://pesquisa.bvsalud.org/bvsms/?lang=pt&fb=&q=%28caffein*+OR+Xanthin*+OR+Methylxanthine%29+AND+%28preterm*+OR+newborn*+OR+neonat*+OR+prematur*%29&skfp=true&range_year_start=&range_year_end=

**Supplementary Figures: Summary of risk of bias evaluation:**


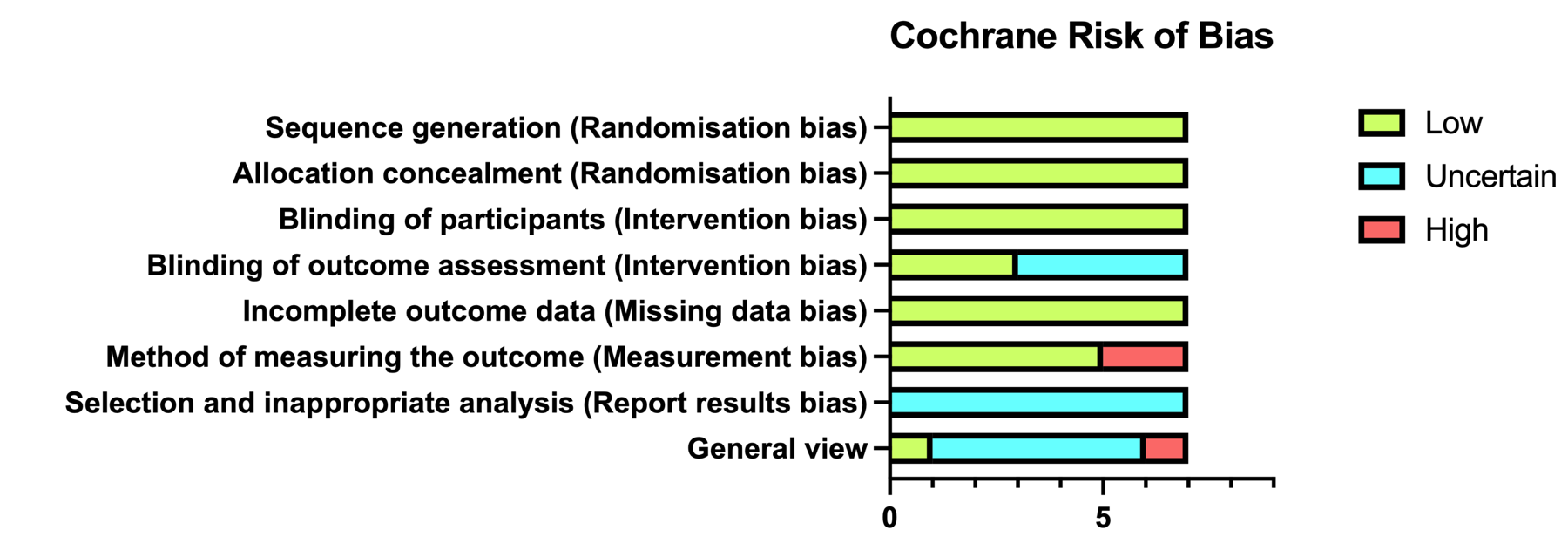


Supplementary figure 1: Summary of Cochrane Risk of Bias analysis following Sterne and colleagues [19].


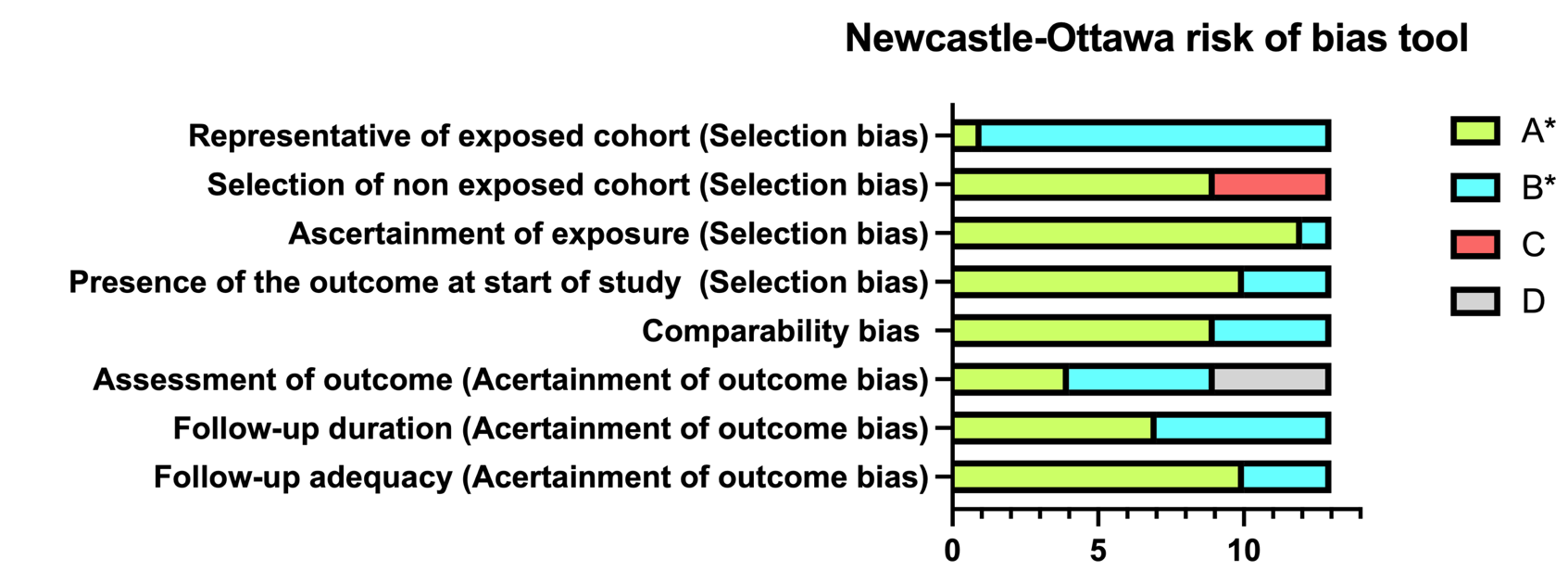


Supplementary figure 2: Summary of Newcastle-Ottawa of Bias analysis. A* and B* = low risk of bias and C and D= high risk of bias following Wells and colleagues [20]
